# Collagen X Is Dispensable for Hypertrophic Differentiation and Endochondral Ossification of Human iPSC‐Derived Chondrocytes

**DOI:** 10.1002/jbm4.10737

**Published:** 2023-03-29

**Authors:** Takeshi Kamakura, Yonghui Jin, Megumi Nishio, Sanae Nagata, Masayuki Fukuda, Liping Sun, Shunsuke Kawai, Junya Toguchida

**Affiliations:** ^1^ Department of Regeneration Sciences and Engineering, Institute for Life and Medical Sciences Kyoto University Kyoto Japan; ^2^ Department of Fundamental Cell Technology, Center for iPS Cell Research and Application Kyoto University Kyoto Japan

**Keywords:** GROWTH PLATE, CHONDROCYTE AND CARTILAGE BIOLOGY, DISEASES AND DISORDERS OF/RELATED TO BONE, COLLAGEN, BONE MATRIX

## Abstract

Collagen X is a non‐fibril collagen produced by hypertrophic chondrocytes and was believed to associate with the calcification process of growth plate cartilage. The homozygous loss of *Col10a1* gene in mice, however, demonstrated no remarkable effects on growth plate formation or skeletal development. To investigate the role of collagen X in human chondrocytes, we established human induced pluripotent stem cells (hiPSCs) with heterozygous (*COL10A1* ^*+/−*^) or homozygous (*COL10A1* ^*−/−*^) deletions of *COL10A1* gene using the dual sgRNA CRISPR/Cas9 system. Several mutant clones were established and differentiated into hypertrophic chondrocytes by a previously reported 3D induction method. No remarkable differences were observed during the differentiation process between parental and mutant cell lines, which differentiated into cells with features of hypertrophic chondrocytes, indicating that collagen X is dispensable for the hypertrophic differentiation of human chondrocytes in vitro. To investigate the effects of collagen X deficiency in vivo, chondrocyte pellets at the proliferating or prehypertrophic stage were transplanted into immunodeficient mice. Proliferating pellet‐derived tissues demonstrated the zonal distribution of chondrocytes with the transition to bone tissues mimicking growth plates, and the proportion of bone tended to be larger in *COL10A1* ^*−/−*^ tissues. Prehypertrophic pellet‐derived tissues produced trabecular bone structures with features of endochondral ossification, and there was no clear difference between parental‐ and mutant‐derived tissues. A transcriptome analysis of chondrocyte pellets at the hypertrophic phase showed a lower expression of proliferating‐phase genes and a higher expression of calcification‐phase genes in *COL10A1* ^*−/−*^ pellets compared with parental cell pellets. These in vitro and in vivo data suggested that collagen X is dispensable for the hypertrophic differentiation and endochondral ossification of human iPSC‐derived chondrocytes, though it may facilitate the differentiation process. Thus, *COL10A1* ^*−/−*^ iPSC lines are useful for investigating the physiological role of collagen X in chondrocyte differentiation. © 2023 The Authors. *JBMR Plus* published by Wiley Periodicals LLC on behalf of American Society for Bone and Mineral Research.

## Graphical abstract

Type X collagen (COL X) is a cartilage matrix protein produced by hypertrophic chondrocytes. Human iPS cells with homozygous deletions of *COL10A1* gene created by a dual sgRNA CRISPR/Cas9 system differentiated into hypertrophic chondrocytes in vitro and demonstrated the formation of growth plate‐like structure and endochondral ossification in vivo, indicating that COL X is dispensable for these biological processes.

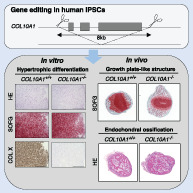

## Introduction

Growth plates are a highly organized cartilaginous structure located between the epiphysis and metaphysis of tubular bones, which play a crucial role in longitudinal bone growth until skeletal maturity.^(^ ^1^, ^2^ ^)^ There are four distinct zones in the growth plate—resting, proliferating, prehypertrophic, and hypertrophic—and chondrocytes in the last three create columnar structures showing gradual morphological changes, in which hypertrophic changes are an important step for the lengthening of a skeletal element.^(^ ^3^ ^)^ Chondrocytes in each zone express zone‐specific signal molecules and produce a zone‐specific extracellular matrix, and the transition between zones is strictly regulated by local and systemic factors. The terminal phase forms calcified matrix and shows vascular invasion, which initiates bone formation. The fate of hypertrophic chondrocytes has been a long‐standing issue, and debate continues about whether they die by apoptosis or undergo osteogenic transdifferentiation.^(^ ^4^ ^)^ Recent lineage tracing studies indicated hypertrophic chondrocytes are a potent source of osteogenic cells.^(^ ^5^, ^6^ ^)^ One specific marker of hypertrophic chondrocytes is collagen X (COL X), which is encoded by *COL10A1* gene. COL X is a homotrimer of three 59 kDa α1(X) chains, with a short (38 aa) global nonhelical amino terminus (NC2), a triple helix of 463 aa, and a carboxyl terminal highly conserved non‐collagenous domain (NC1) of 161 aa. It does not form fibrils but assembles into a hexagonal lattice that associates with fibrils of other collagens by forming pericellular filaments.^(^ ^7^, ^8^ ^)^ In addition, COL X interacts with other matrix components such as proteoglycan and binds to matrix vesicles,^(^ ^9^ ^)^ which serve as seeds for the initiation of mineral deposition. *COL10A1* gene is expressed exclusively in hypertrophic chondrocytes, suggesting its importance in calcification and ossification in the growth plate.

Therefore, the results of a knockout mice study nearly 30 years ago were a great surprise, as the mice showed no overt abnormalities in skeletal development or the apparent structure of the growth plate.^(^ ^10^ ^)^ This model argued that the expression of *COL10A1* is not a causal factor for chondrocyte hypertrophy but rather a result of hypertrophic differentiation. Further analysis of another knockout mouse model, however, demonstrated that an abnormal reduction in the thickness of the growth plate resting zone and articular cartilage altered bone content and led to an atypical distribution of matrix components within growth plate cartilage, indicating that the loss of COL X has an effect on chondrocyte differentiation.^(^ ^11^ ^)^ It is not known whether the discrepancy between the two reports is attributable to the difference in analytical methods or genomic structures of the knockout *Col10a1* locus.

Loss‐of‐function mutations of *COL10A1* gene were reported in a hereditary skeletal disease, Schmid‐type metaphyseal chondrodysplasia (SMCD),^(^ ^12^ ^)^ which is characterized by moderate short limbs and short stature and a waddling gait caused by bowed legs.^(^ ^13^ ^)^ A large number of different mutations have been reported, and almost all of them were found in NC1 domain.^(^ ^14^ ^)^ Half of cases were caused by missense mutations producing mutant α1(X), which interferes with normal α1(X) to form a triple helix. In other cases, mutations produce a premature stop codon and no mRNA transcribed from mutant alleles due to nonsense mediated decay, suggesting that haploinsufficiency was a pathogenic mechanism of this disease.^(^ ^15^, ^16^ ^)^ Other studies, however, demonstrated that both mutant and wild‐type mRNA were equally transcribed,^(^ ^17^ ^)^ and several in vitro reports demonstrated that those mutations produced truncated peptides that interfere with the triple helix formation, exerting a gain‐of‐function effect as well as missense mutations.^(^ ^18^, ^19^ ^)^ SMCD model mice with nonsense and frameshift mutations also showed gain‐of‐function effects that activated the unfolded protein response (UPR).^(^ ^20^ ^)^ Therefore, whether a loss of COL X has any effect on the differentiation process of chondrocytes is still to be investigated, particularly in human samples.

Recently, we established a method to induce hypertrophic chondrocytes from human induced pluripotent stem cells (hiPSCs) and made growth plate‐like structures in mice by transplanting the cells.^(^ ^21^, ^22^ ^)^ Following those studies, here we established human iPSCs with the heterozygous or homozygous loss of *COL10A1* gene and induced hypertrophic differentiation in the cells. The results indicated that the loss of COL X does not affect hypertrophic differentiation or endochondral bone formation, but it may modulate the differentiation process of chondrocytes induced from hiPSCs.

## Materials and Methods

### Gene editing

Two hiPSC lines (414C2 and 1231A3) established at our institute^(^ ^23^, ^24^ ^)^ were used to generate COL10A1 knockout hiPSCs using the dual sgRNA CRISPR/Cas9 system.^(^ ^25^, ^26^ ^)^ Dual sgRNAs were designed to target the N‐terminal and C‐terminal intergenic regions of *COL10A1* gene and inserted into pSpCas9(BB)‐2A‐Puro (PX459) V2.0 vector (Addgene, Watertown, MA, USA) using Ligation high Ver.2 (TOYOBO, Osaka, Japan) as previously described.^(^ ^27^ ^)^ The vectors were transfected into feeder‐free parental hiPSCs using lipofectamine stem transfection reagent (Invitrogen, Carlsbad, CA, USA) and Opti‐MEM (Gibco, Thermo Fisher Scientific, Waltham, MA, USA) following the manufacturer's protocol. Cells were selected with 0.3 μg/mL puromycin (Gibco) for 72 hours to enrich a population of gene‐edited cells, and then single colonies were randomly picked 10 days after sparsely reseeding the cells. Genomic DNA was extracted using the DNeasy Blood and Tissue Kit (QIAGEN, Valencia, CA, USA) with proteinase K and RNase A treatment, and then relevant regions were amplified by PCR using KOD One PCR Master Mix (TOYOBO). Amplicons were purified using the FastGene Gel/PCR Extraction Kit (Nippon Genetics, Tokyo, Japan) after agarose gel electrophoresis and sequenced using the BigDye Terminator v3.1 Cycle Sequencing Kit (Applied Biosystems, Carlsbad, CA, USA) with a 3500xL Genetic Analyzer (Applied Biosystems). The primers used are listed in Supplemental Table S1.

### Culture and validation of gene‐edited hiPSCs

Gene‐edited hiPSC clones were expanded and maintained the same way as parental hiPSCs, which were maintained feeder‐free on dishes coated with iMatrix‐511 silk (nippi) in StemFit AK02N (Ajinomoto, Tokyo, Japan) with 50 U of penicillin and 50 μg/mL streptomycin (Gibco) and passaged as single cells once a week at a density of 1.5 × 10^3^ cells/well in 6‐well plates. Karyotype analysis was performed using the Q‐banding method by Chromocenter Inc. (Tottori, Japan). Differentiation properties for the three germ layers were confirmed by teratoma formation. An amount of 1 × 10^6^ cells were injected subcutaneously into at least three NOD‐SCID mice, which were euthanized 10 to 12 weeks later to excise the tissues. After fixation in 4% paraformaldehyde (PFA) overnight at 4°C, the tissues were embedded in paraffin, sectioned, and stained with hematoxylin–eosin (HE).

### Induction of hypertrophic chondrocytes

Hypertrophic chondrocytes were induced from hiPSC via sclerotome as previously described (Supplemental Fig. S2).^(^ ^21^, ^22^ ^)^ Briefly, hiPSCs were cultured in chemically defined medium with insulin (CDMi) consisting of BSA (5 mg/mL, Sigma, St. Louis, MO, USA), CD Lipid (1% [v/v], Gibco), apo‐transferrin (15 μg/mL, Sigma), monothioglycerol (450 μM, Sigma), penicillin (50 U), streptomycin (50 μg/mL), and insulin (7 μg/mL, Wako, Osaka, Japan) in 1:1 mixture of Iscove's modified Dulbecco's medium (IMDM) (Sigma) and Ham's F12 (Gibco). Primitive streak cells were induced from hiPSCs using CDMi with bFGF (20 ng/mL, Wako), CHIR99021 (10 μM, Axon, Groningen, the Netherlands), and Activin A (50 ng/mL, R&D Systems, Minneapolis, MN, USA), followed by presomitic mesoderm induction with bFGF (20 ng/mL), CHIR99021 (3 μM), SB431542 (10 μM, Selleck Chemicals, Houston, TX, USA) and LDN193189 (250 nM, Stemgent, Reprocell, Yokohama, Japan), somitic mesoderm induction with PD173074 (100 nM, Tocris, Bristol, UK) and XAV939 (1 μM, Tocris), and lastly sclerotome induction with LDN193189 (600 nM) and SAG (100 nM, Calbiochem, MilliporeSigma, Burlington, MA, USA). Sclerotome cells were then seeded into low‐attachment 96‐well plates at 2.5 × 10^5^ cells/well. Hypertrophic chondrocyte induction was initiated by chondrogenic medium consisting of ITS premix (1% [v/v], Corning Inc., Corning, NY, USA), L‐ascorbic acid 2‐phosphate (170 μM, Sigma), proline (350 μM, Sigma), glucose (0.15% [v/v], Sigma), sodium pyruvate (1 mM, Sigma), GlutaMAX (2 mM, Gibco), penicillin (100 U), and streptomycin (100 μg/mL) in DMEM/F12 (Gibco) supplemented with PDGF‐BB (40 ng/mL) and dexamethasone (100 nM, Wako), followed by the addition of TGFβ3 (10 ng/mL, R&D Systems), replacement of PDGF‐BB with BMP4 (50 ng/mL, R&D Systems), replacement of TGFβ3 with triiodothyronine (10 nM, Sigma), and finally the addition of β‐glycerophosphate (10 mM, Sigma).

### Flow cytometry analysis

Presomitic mesoderm cells were detached and washed with FACS buffer containing 0.1% (w/v) BSA in PBS (−). Approximately 5 × 10^5^ cells were stained with either APC‐conjugated DLL1 antibody (R&D Systems) or APC‐conjugated mouse IgG2B (R&D Systems) as an isotype at 1:200 dilution in FACS buffer for 30 minutes at 4°C. After washing two times with FACS buffer, the cells were stained with DAPI (1 μg/mL, R&D Systems) to label dead cells. The analysis was performed using a FACS Aria II and FACSDiva software (BD Biosciences, San Jose, CA, USA).

### mRNA expression analysis

Three‐dimensional chondrocyte pellets were transferred to microtubes, washed with PBS (−) and then quickly frozen with liquid nitrogen. Frozen pellets were homogenized with a Multi‐beads shocker (Yasui Kikai, Osaka, Japan) and lysed with RLT buffer (QIAGEN). Total RNA was extracted using the RNeasy Micro Kit or RNeasy Mini Kit (QIAGEN) with DNase I treatment. cDNA was synthesized using ReverTra Ace (TOYOBO) in a total volume of 20 μL with up to 500 ng of RNA. Synthesized cDNA was used for quantitative real‐time PCR (qRT‐PCR) using THUNDERBIRD SYBR qPCR Mix (TOYOBO) and with QuantStudio 12 K Flex Real‐Time PCR System (Applied Biosystems). The primers used are listed in Supplemental Table S2.

### Protein expression analysis

Three‐dimensional chondrocyte pellets were transferred to microtubes, washed with PBS (−) and stored at −80°C. After adding 100 μL RIPA buffer (Nacalai Tesque, Kyoto, Japan) with 1% (v/v) protease inhibitor cocktail (Nacalai Tesque), pellets were homogenized on ice until complete dissolution. Homogenized samples were centrifuged, and supernatants were collected. The protein concentration was measured using a Pierce BCA Protein Assay Kit (Thermo Fisher Scientific) with an EnVision 2104 Multi plate reader (PerkinElmer, Waltham, MA, USA). The expression of each protein was detected using a 12–230 kDa or 60–440 kDa Separation Module with Wes (ProteinSimple, Minneapolis, MN, USA) automated capillary‐based immunoassay system called “Simple Western.” For each lane, 2 μg of protein at a concentration of 0.5 μg/μL, primary antibodies, and Anti‐Rabbit or Anti‐Mouse Detection Module (ProteinSimple) were loaded and ran according to the manufacturer's instructions. Antibodies were diluted with Antibody diluent II (ProteinSimple) to 1:100 for β‐actin (CST, #4970) and COL X (Abcam, Cambridge, UK; ab182563) and 1:50 for COL I (Novus, Littleton, CO, USA; NBP1‐30054) and COL II (Novus, NB600‐844).

### Histological analysis

Three‐dimensional chondrocyte pellets were transferred to microtubes, washed with PBS (−), and fixed with 4% PFA overnight at 4°C. Fixed pellets were embedded in paraffin and sectioned with microtome. The sections were deparaffinized and stained with HE or Safranin‐O and Fast‐Green (SOFG). For immunohistochemistry, the sections were incubated with EDTA for 20 minutes at 98°C or with proteinase K for 5 minutes at room temperature for antigen retrieval and then incubated with 3% BSA for blocking. The sections were incubated overnight at 4°C with antibodies against COL I (SBA, 1310–01) at 1:500, COL II (SBA, 1320–01) at 1:600, COL X (Invitrogen, 14–9771‐82) at 1:400, or HNA (MilliporeSigma) at 1:250. They were then stained with Simple Stain MAX‐PO (G) or (M) (Nichirei, Tokyo, Japan) to detect the signals and observed with an All‐in‐One Fluorescence Microscope BZ‐X800 (KEYENCE, Osaka, Japan).

### Animal experiments

Three‐dimensional chondrocyte pellets at day 14 or 28 were transplanted subcutaneously into at least three female immunodeficient NOD/ShiJic‐scidJcl (NOD‐SCID) mice (CLEA Japan, Tokyo, Japan) as previously described.^(^ ^22^ ^)^ Ossification of the transplants was monitored using an X‐ray imaging device DX‐50 (Faxitron, Tucson, AZ, USA) biweekly. Animal experiments were approved by the institutional animal committee of Kyoto University and performed in strict accordance with the Regulation on Animal Experimentation at Kyoto University.

### Image quantification

Sections of in vitro 3D pellets and in vivo tissues were viewed with a BZ‐X800 microscope and then analyzed with BZ‐X800 Analyzer software (KEYENCE). High‐resolution whole images of in vivo tissues were created by seamlessly stitching 30 images at 10× magnification using Multistack Module (KEYENCE). The COL II‐positive area (μm^2^) was delineated and quantified using Hybrid Cell Count Module (KEYENCE), and the ratio to whole tissue area (WTA) (μm^2^) was calculated.

### RNA sequencing

A library was made from 100 ng of total RNA extracted from 3D chondrocyte pellets at day 42 using TruSeq Stranded Total RNA Library Prep Gold (Illumina, San Diego, CA, USA). Sequencing was performed as paired‐end, 101 bp reads using the NovaSeq 6000 SP Reagent Kit v1.5 (200 cycles) with NovaSeq 6000 (Illumina). The sequencing depth was 50 million reads per sample. Processing and differentially expressed gene (DEG) analysis were performed as previously described.^(^ ^28^, ^29^ ^)^ In brief, the sequenced reads were mapped to the human reference genome GRCh38 v105 using STAR 2.5.1b^(^ ^30^ ^)^ with GENCODE v39 gene annotations, and the expected gene counts were calculated and normalized with transcripts per kilobase million (TPM) using RSEM 1.2.23.^(^ ^31^ ^)^ DEG analysis was performed using DESeq2 1.34.0 (Bioconductor) as previously described,^(^ ^32^ ^)^ and commonly up‐ or downregulated genes among homozygous knockout clones were visualized with Venn diagrams and tables. A principal component analysis (PCA) was plotted based on gene expression profiles normalized with a variance stabilizing transformation (VST). The library preparation, sequencing, processing, and DEG analysis were performed by the CiRA Foundation (Kyoto, Japan).

### Statistical analysis

Statistical analyses and graph generations were performed using Prism v9.4.1 (GraphPad Software, La Jolla, CA, USA). Data are presented as boxplots with the minimum and maximum in each figure. Statistical significance was determined by a one‐way or two‐way ANOVA with multiple comparisons test as specified in each figure. Exact *p* values are represented as 0.0001 < *p* < 0.1.

## Results

### Establishment of human iPSCs lacking *COL10A1* genes

**Fig. 1:**
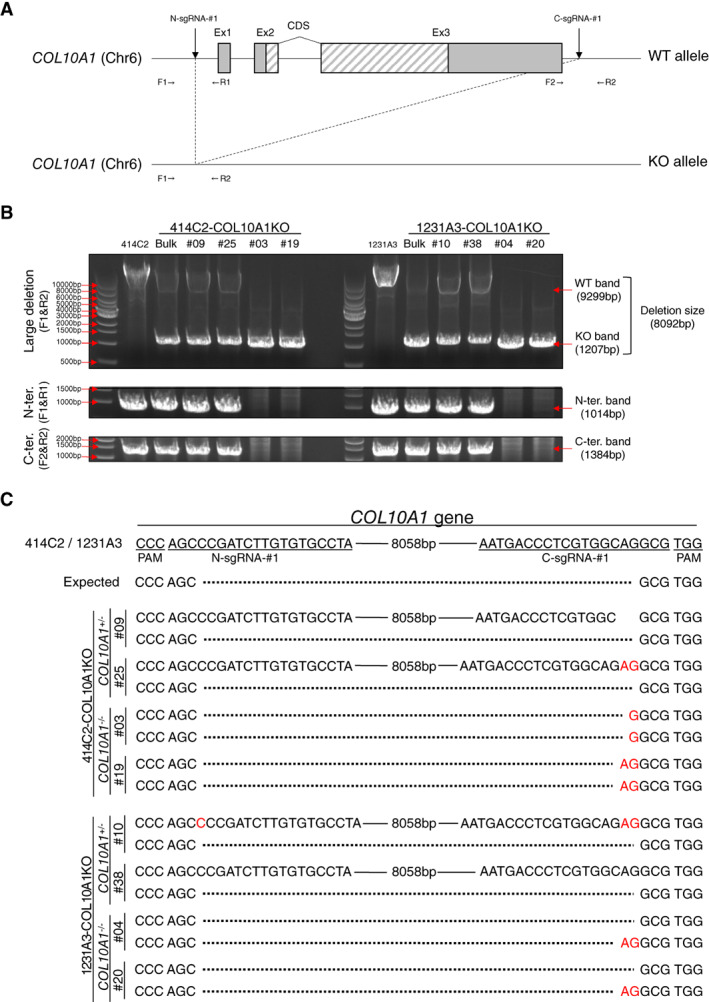
Generation of COL10A1‐KO cell lines from human iPS cell lines. (*A*) Schematic diagram of the *COL10A1* locus before (upper) and after (lower) genome editing using N‐ and C‐sgRNA. Gray and hatched boxes indicate noncoding and coding regions, respectively. F1, F2, R1, and R2 indicate the location of PCR primers for the evaluation of the genome editing. (*B*) Demonstration of an 8.1 kb deletion in the *COL10A1* locus by genomic DNA PCR. Deletion is confirmed by the loss of a longer fragment (9.3 kb). Shorter fragments from N‐terminal and C‐terminal regions are amplified in cases with a heterozygous deletion but not with homozygous deletions. (*C*) Sequences of the longer and shorter fragments demonstrated in (*B*). Solid and dotted lines indicate conserved and deleted regions, respectively. Inserted sequences are highlighted in red.

To avoid the effect of truncated peptides produced by a premature stop codon, two gRNAs, N‐sgRNA and C‐sgRNA, were designed to delete the entire coding regions of *COL10A1* gene using the dual CRISPR/Cas9 system (Fig. 1*A*). Each fragment was cloned into the expressing vector and transfected to 414C2 or 1231A3 cells using lipofection as described in Materials and Methods. After the drug selection, several single cell–derived clones were isolated, the structure of the *COL10A1* locus was analyzed by genomic PCR and sequencing, and clones for further analysis were selected (Fig. 1*B*, *C*).

PCR primers were designed to amplify 9299 bp fragments from wild‐type loci and 1207 bp fragments from precisely deleted loci (Fig. 1*B*). 414C2‐derived #09 and #25 clones and 1231A3‐derived #10 and #38 clones showed both longer and shorter fragments, suggesting heterozygous deletion (Fig. 1*B*). A sequencing analysis of shorter fragments showed a complete match with the predicted deletion in all clones (Fig. 1*C*). A sequencing analysis of longer fragments around the target region demonstrated no alterations (#38), short deletions (#09), or insertions (#25 and #10) in either end, but no other alterations were found in other regions, suggesting the preservation of the wild‐type allele. We used these four clones as heterozygously knockout clones (*COL10A1* ^+/−^ clones).

Clones showing no longer fragments (414C2‐derived #03 and #19 clones and 1231A3‐derived #04 and #20 clones) (Fig. 1*B*) were considered to have rearrangements in both alleles. A sequence analysis of shorter fragments showed a single sequence with short insertions at the junction site (#03 and #19), which indicated identical events occurred in the two alleles (Fig. 1*C*). On the other hand, other clones (#04 and #20) showed a mixture of two different sequences, each of which showed no alterations or short insertions at the junction site (Fig. 1*C*). These data indicated that different genomic rearrangements occurred in the two alleles. We used these four clones as homozygously knockout clones (*COL10A1* ^−/−^ clones).

The cellular morphology of *COL10A1* ^+/−^ or *COL10A1* ^−/−^ iPSC clones showed no remarkable differences from those of the parental cell lines (Supplemental Fig. S1*A*). A karyotype analysis also demonstrated no difference between parental cell lines (Supplemental Fig. S1*B*), and the pluripotency of these clones was confirmed by teratoma formation (Supplemental Fig. S1*C*).

### COL X is dispensable for hypertrophic differentiation of chondrocytes induced from human iPSCs

**Fig. 2:**
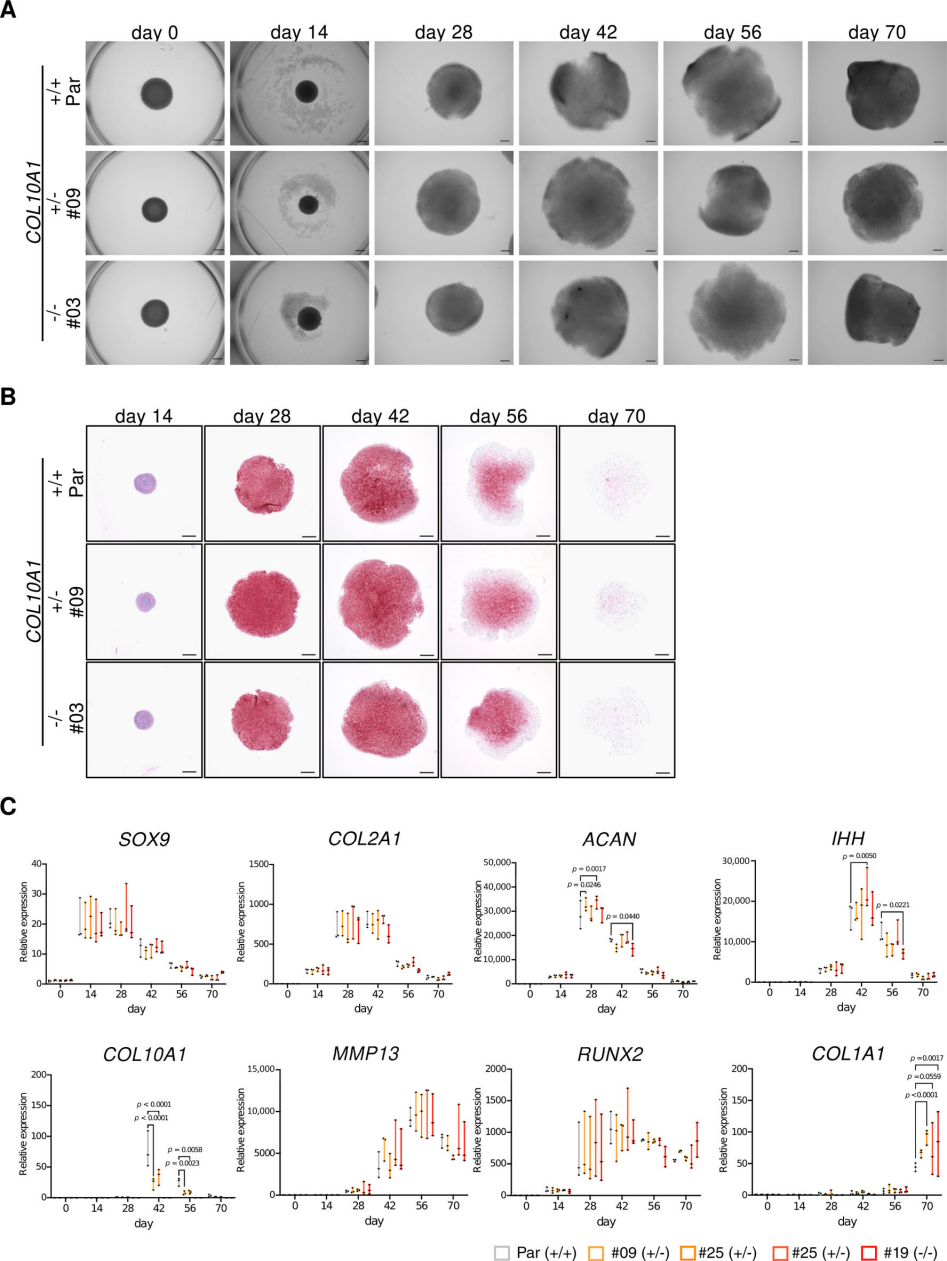
Induction of hypertrophic chondrocytes from parental (414C2), *COL10A1* ^+/−^, and *COL10A1* ^−/−^ iPSC lines. (*A*) Phase contrast images and (*B*) Safranin‐O and Fast‐Green staining of cell pellets at different days during the induction. Scale bar = 500 μm. Similar results were obtained in three independent experiments. (*C*) mRNA expression of growth plate–related genes during the induction. RNAs were extracted from 3D chondrocyte pellets at each time point and assessed for the expression of each gene by qRT‐PCR. The expression level was normalized to that of parental iPSC‐derived pellets at day 0, except *COL10A1*, for which the level at day 28 was used for the normalization. Statistical analysis was performed using a two‐way ANOVA. Data are presented as boxplots presenting all points from the minimum to maximum values (*n* = 3, independent experiments).

*COL10A1* ^+/−^ and *COL10A1* ^−/−^ iPSC clones as well as the parental cells were differentiated to hypertrophic chondrocytes using 3D induction (Supplemental Fig. S2), and morphological, histological, and molecular profiles during the differentiation were sequentially monitored (Fig. 2 and Supplemental Fig. S3). After day 14, cellular pellets gradually enlarged until day 42 and then remained a constant size (Fig. 2*A* and Supplemental Fig. S3*A*). A Safranin‐O‐positive area emerged from the periphery and reached its peak at day 42, but then the peripheral regions gradually lost their staining (Fig. 2*B* and Supplemental Fig. S3*B*). The cellular morphology gradually changed, and the size of the cells in the peripheral regions was larger than those in the central regions. These sequential changes showed no obvious differences among parental, *COL10A1* ^+/−^, and *COL10A1* ^−/−^ iPSC clones. The expression of growth plate–related genes was sequentially monitored (Fig. 2*C* and Supplemental Fig. S3*C*). The expression level of *COL10A1* gene was less than 50% in *COL10A1* ^+/−^ iPSC clones, suggesting no compensatory upregulation of the remaining allele. No other genes showed constant differences among parental and mutant clones except the expression of *COL1A1* at day 70, which was elevated in *COL10A1* ^−/−^ iPSC clones.

**Fig. 3:**
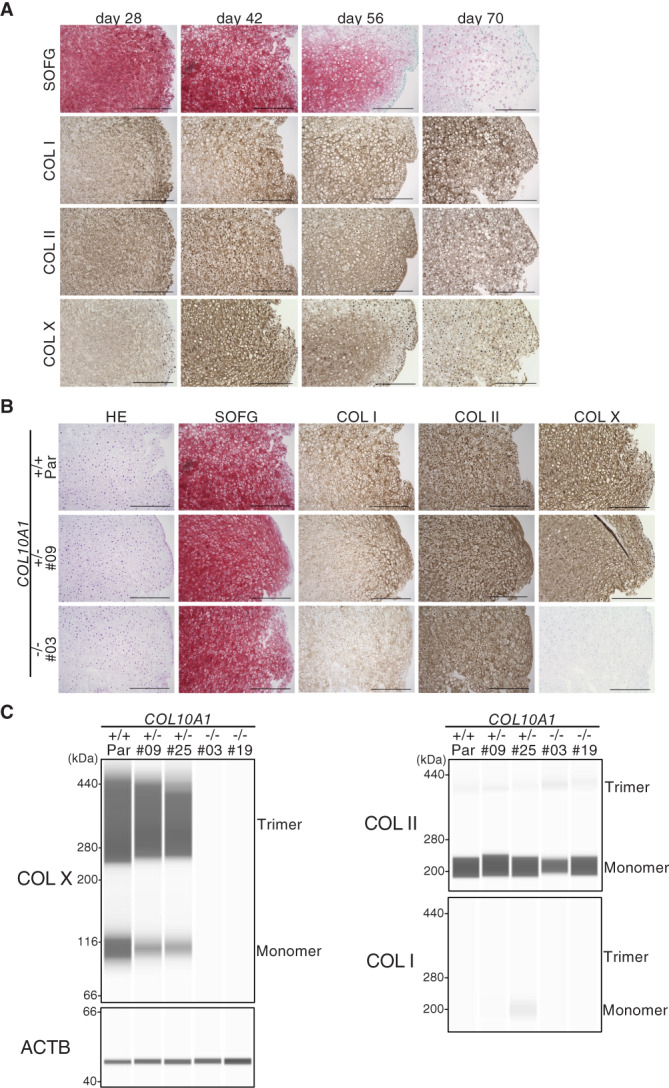
Expression of cartilage matrix at day 42 in parental (414C2), *COL10A1* ^+/−^, and *COL10A1* ^−/−^ iPSC‐derived 3D chondrocyte pellets. (*A*) Histological evaluation of parental iPSC‐derived pellets. Cell pellets at each time point were stained with Safranin‐O and Fast‐Green (SOFG) and antibodies against COL I, COL II, or COL X. Scale bar = 500 μm. (*B*) Histological evaluation of parental, *COL10A1* ^+/−^, and *COL10A1* ^−/−^ iPSC‐derived pellets at day 42. Cell pellets were stained with SOFG and antibodies against COL I, COL II, or COL X. Scale bar = 500 μm. (*C*) Capillary‐based immunoassay of proteins extracted from cell pellets. Cellular proteins were extracted from pellets at day 42 and analyzed by Western blot using antibodies against COL I, COL II, or COL X. The experiments were performed three times with similar results.

The expression of COL I, COL II, and COL X protein were sequentially analyzed during the induction of parental iPSCs (Fig. 3*A* and Supplemental Fig. S4*A*). The Safranin‐O‐positive area gradually decreased from day 28 to day 70, and COL II staining was also diminished during the culture period. On the contrary, COL I staining peaked at day 70 and COL X staining at day 42. Based on these data, the expression of these collagens in mutant cells was analyzed at day 42 by immunohistochemical staining and also by Western blot (Fig. 3*B*, *C* and Supplemental Fig. S4*B, C*). Both analyses showed the reduction of COL X in *COL10A1* ^+/−^ clones and no expression in *COL10A1* ^−/−^ clones, confirming the effect of genome editing. No remarkable difference was found in the data of COL I and COL II between parental and mutant cell lines. These data indicated that COL X is dispensable for the in vitro hypertrophic differentiation of human chondrocytes induced from iPSCs.

### COL X is dispensable for the formation of growth plate‐like structures and trabecular bone formation

**Fig. 4:**
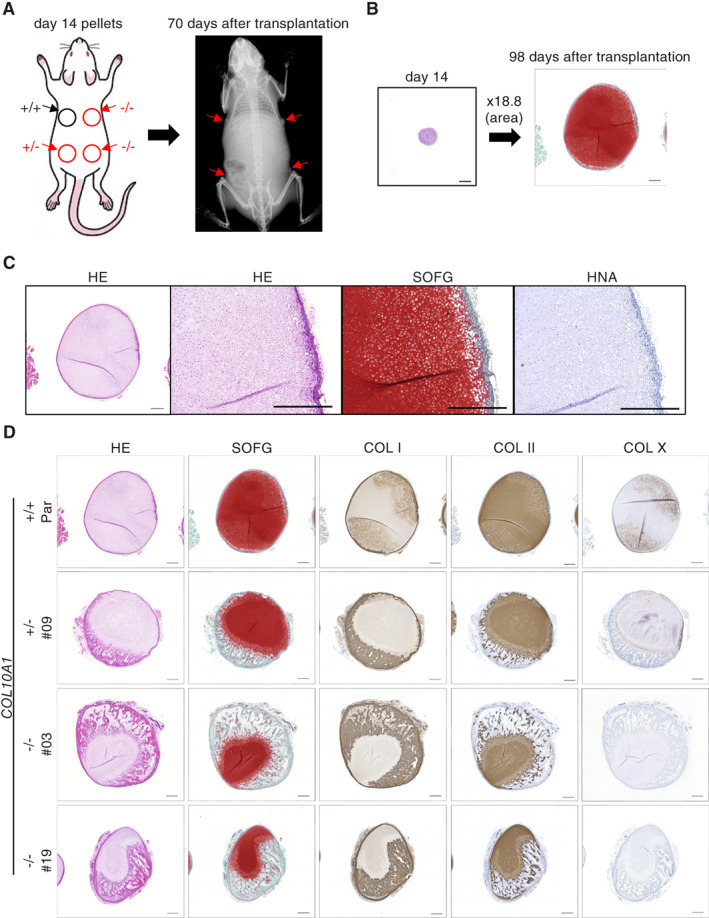
Evaluation of chondrocyte pellets at the proliferating stage in vivo. (*A*) Cell pellets derived from parental (414C2), *COL10A1* ^*+/−*^, and *COL10A1* ^*−/−*^ iPSC lines at day 14 were transplanted into immunodeficient mice, and ossification in the transplants was analyzed by X‐ray biweekly. (*B*) Comparison between original pellets and samples collected 98 days after the transplantation. The size of the cutting surface area was enlarged 18.8 times. Pellets were stained with Safranin‐O and Fast‐Green. Scale bar = 500 μm. (*C*) Histology of parental iPSC‐derived transplants. The collected samples were stained with hematoxylin–eosin (HE), Safranin‐O and Fast‐Green (SOFG), or an antibody against human nuclear antigen (HNA). Scale bar = 500 μm. (*D*) Histology of parental, *COL10A1* ^*+/−*^, and *COL10A1* ^*−/−*^ iPSC‐derived transplants. The collected samples were stained with HE, SOFG, and antibodies against COL I, COL II, or COL X. Scale bar = 500 μm. Similar results were obtained in three independent experiments.

To investigate the effect of the loss of COL X in vivo, we performed two types of transplantation experiments (Supplemental Fig. S2). One was to transplant 3D pellets at day 14 (proliferating stage) and collected 98 days later after confirming the ossification of the transplants at day 70 by X‐ray images (Fig. 4*A*). Based on the size of the section area, the volume of the collected tissues was almost 80 times larger than that of the initial pellets, indicating the vigorous growth potential of the cells at day 14 (Fig. 4*B*). Histological examination of the collected tissues derived from the parental pellets showed uni‐ or bidirectional zonal distribution of the chondrocytes with bone tissues mimicking growth plates (Fig. 4*C* and Supplemental S5 *A*). The formation of these structures was also observed in tissues derived from mutant pellets (Fig. 4*D* and Supplemental Fig. S5*B*). The bone area was positive for COL I in all tissues, and COL X was stained around hypertrophic chondrocytes in parental and *COL10A1* ^+/−^ pellet‐derived tissues (Fig. 4*D* and Supplemental Fig. S5*B*). In the case of 414C2‐derived tissues, the proportion of the COL II‐positive area in *COL10A1* ^−/−^ tissues was smaller than in parental tissues (Supplemental Fig. S6*A*). In other words, the bone area was larger in *COL10A1* ^−/−^ tissues. Such differences were not clearly observed in 1231A3‐derived tissues (Supplemental Fig. S6*B*).

**Fig. 5:**
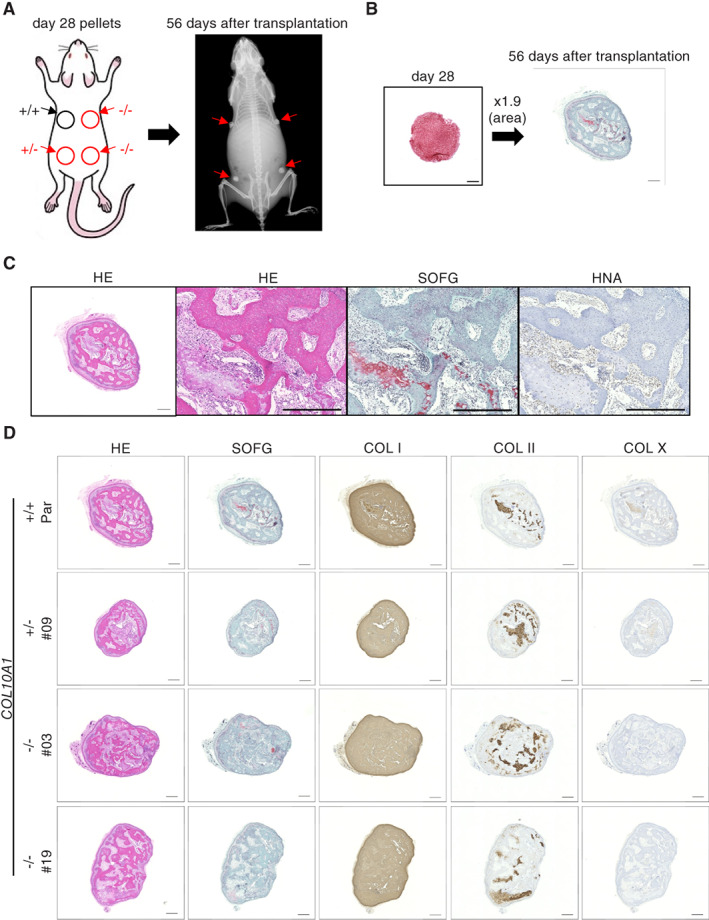
Evaluation of chondrocyte pellets at the prehypertrophic stage in vivo. (*A*) Cell pellets derived from parental (414C2), *COL10A1* ^*+/−*^, and *COL10A1* ^*−/−*^ iPSC lines at day 28 were transplanted into immunodeficient mice, and ossification in the transplants was analyzed by X‐ray biweekly. (*B*) Comparison of original pellets and samples collected 56 days after the transplantation. The size of the cutting surface area was enlarged 1.9 times. Pellets were stained with Safranin‐O and Fast‐Green. Scale bar = 500 μm. (*C*) Histology of parental iPSC‐derived transplants. The collected samples were stained with hematoxylin–eosin (HE), Safranin‐O and Fast‐Green (SOFG), or an antibody against human nuclear antigen (HNA). Scale bar = 500 μm. (*D*) Histology of parental, *COL10A1* ^*+/−*^, and *COL10A1* ^*−/−*^ iPSC‐derived transplants. The collected samples were stained with HE, SOFG, and antibodies against COL I, COL II, or COL X. Scale bar = 500 μm. Similar results were obtained in three independent experiments.

The other experiment was to transplant 3D pellets at day 28 (prehypertrophic stage) and collect them 56 days later, when bone tissue formation was confirmed by X‐ray images (Fig. 5*A*). The volume of the collected tissues was only 2.6 times larger than that of the initial pellets (Fig. 5*B*). A histological examination of tissues derived from parental pellets showed random trabecular bone formation with immature chondroid tissues, featuring the endochondral ossification process (Fig. 5*C* and Supplemental S7*A*). Cells embedded in bones and lining cells were HNA positive, and stromal cells among trabeculae were a mixture of positive and negative cells (Fig. 5*C* and Supplemental S7*A*). The amount and structure of trabecular bones and the distribution of the COL I‐ and COL II‐positive area showed no clear differences between samples derived from parental and mutant cell lines (Fig. 5*D* and Supplemental S7*B*). Only a small area was positive for COL X in parental and *COL10A1* ^*+/−*^ pellet‐derived tissues. These data indicated that the loss of COL X has little effect on trabecular bone formation.

### Transcriptome analysis disclosed the effect of COL X deficiency in chondrocyte differentiation

**Fig. 6:**
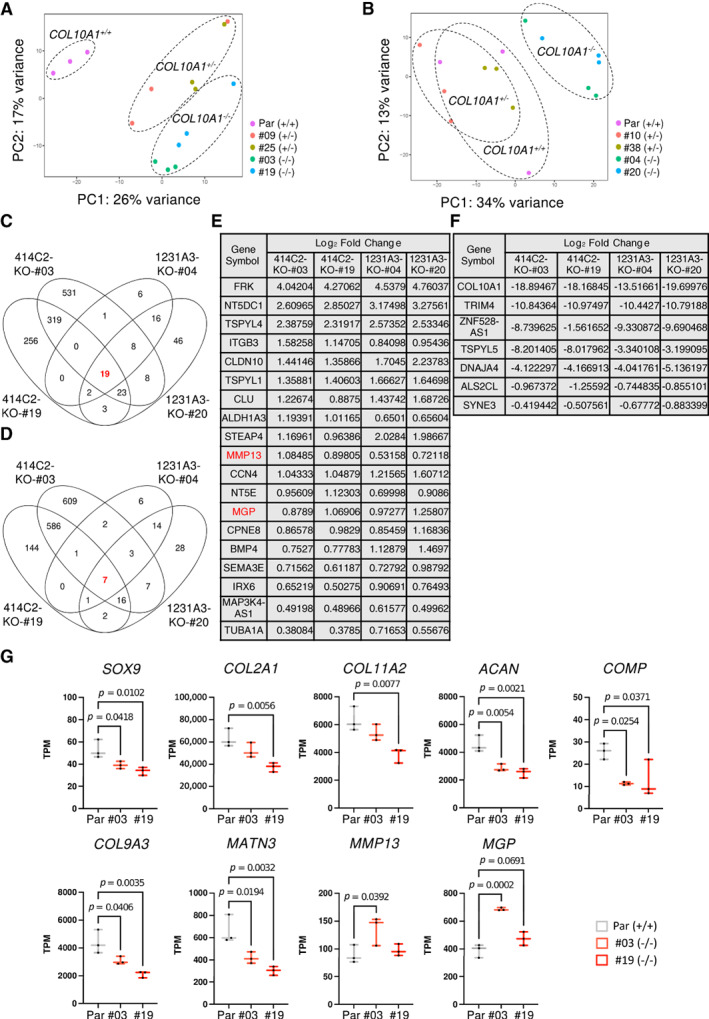
Comparison of mRNA expression profiles between parental and mutant iPSC‐derived 3D chondrocyte pellets at day 42. RNAs were extracted from parental, *COL10A1* ^*+/−*^, and *COL10A1* ^*−/−*^ iPSC‐derived pellets at day 42 and processed for RNA sequencing analyses. (*A*, *B*) PCA of 414C2‐derived cells (*A*) and 1231A3‐derived cells (*B*) (*n* = 3, independent experiments). (*C*, *D*) Venn diagrams showing the overlap of DEGs for up‐ (*C*) and downregulated (*D*) genes in *COL10A1* ^*−/−*^ pellets. (*E, F*) The list of up‐ (*E*) and downregulated (*F*) genes in *COL10A1* ^*−/−*^ pellets. (*G*) Comparison of mRNA expression levels of growth plate–related genes between parental (414C2) and *COL10A1* ^*−/−*^‐derived pellets. Expression levels of each gene are shown as TPM (transcript per million). Statistical analysis was performed using a one‐way ANOVA. Data are represented as boxplots presenting all points from the minimum to maximum (*n* = 3, independent experiments).

In vivo experiments suggested that COL X deficiency might affect the differentiation process from proliferating to hypertrophic chondrocytes. To investigate the effects of COL X deficiency more intensively, the entire expression profiles of chondrocytes derived from mutant clones at day 42 were compared with those from parental iPSCs. A PCA showed clear differences between chondrocytes derived from parental and *COL10A1* ^−/−^ iPSCs (Fig. 6 *A*, *B*). However, no GO terms showed significant differences between the two groups. Then the expression level of individual genes was compared to identify commonly up‐ or downregulated genes in *COL10A1* ^*−/−*^ iPSC‐derived pellets (Fig. 6 *C*, *D*). Nineteen and six genes were identified as up‐ and downregulated genes, respectively (Fig. 6*E*, *F*). Among the upregulated genes were *MGP* and *MMP13* genes, which are involved in the calcification stage of the growth plate.^(^ ^33^, ^34^ ^)^ A comparison of the expression level of growth plate–related genes between parental and *COL10A1* ^*−/−*^ iPSC‐derived pellets was then done (Fig. 6*G* and Supplemental Fig. S8). Although clonal variations seemed present, the expression level of genes related to proliferating chondrocytes, particularly *COMP*, *COL9A3*, and *MATN3*, were lower in *COL10A1* ^*−/−*^ iPSC‐derived pellets than in parental iPSC‐derived pellets, whereas those related to calcification, such as *MGP* and *MMP13*, were higher in *COL10A1* ^*−/−*^ iPSC‐derived pellets than in parental iPSC‐derived pellets. These results, in combination with the in vivo data, suggested that COL X deficiency facilitates the hypertrophic differentiation of chondrocytes induced from human iPSCs.

## Discussion

Here we provide in vitro and in vivo evidence that COL X is dispensable in the hypertrophic differentiation of human chondrocytes and endochondral ossification and that its loss facilitates the differentiation of proliferating to hypertrophic chondrocytes.

The precise function of COL X remains controversial. Because of its specific localization in the hypertrophic zone of the growth plate, several functions have been proposed, including facilitating the removal of cartilage matrix by MMPs, providing space for the deposition of bone matrix, and regulating the mineralization process.^(^ ^35^ ^)^ *COL10A1* gene is evolutionally conserved from osteichthyans, and chondrichthyes have tandemly duplicated *Col10a1* gene copies showing conserved genomic synteny with osteichthyans *Col10a1* gene.^(^ ^36^ ^)^ This evolutionary conservation suggests the important role of COL X in calcification. The apparently normal features of COL X‐deficient mice, however, indicate that COL X is dispensable for calcification. In the present study, we prepared human iPSCs with the homozygous loss of *COL10A1* gene and tested iPSC‐derived hypertrophic chondrocytes in in vitro and in vivo assay systems. The findings, the first for human cells, are consistent with those of knockout mice.

We have performed a number of studies using patient‐derived iPSCs from hereditary skeletal disorders, including those from patients with SMCD.^(^ ^21^, ^22^, ^37^, ^38^ ^)^ The mutation of *COL10A1* gene in one SMCD patient whom we analyzed was an out‐of‐frame deletion in the NC1 domain.^(^ ^22^ ^)^ Hypertrophic chondrocytes induced from iPSCs of this patient showed an accumulation of COL X peptides in the endoplasmic retinaculum (ER) and upregulation of ER stress markers, suggesting that the phenotype of this case is not caused by haploinsufficiency but UPR induced by truncated COL X peptides.^(^ ^22^ ^)^ To knock out the *Col10a1* gene, both of two reports created a premature stop codon by the insertion of a neomycin‐resistant cassette in exon 3, which might produce truncated peptides.^(^ ^10^, ^11^ ^)^ To avoid the production of truncated peptides by nonsense mutations, we deleted the entire coding region of *COL10A1* gene using the dual gRNA CRISPR/Cas9 system, which was reported to efficiently delete genomic regions longer than 50 kb.^(^ ^25^, ^26^ ^)^ Cells with features of hypertrophic chondrocytes were induced from *COL10A1* ^+/−^ and *COL10A1* ^−/−^ iPSCs, and no increase of UPR‐related genes was observed in these cells (data not shown), further indicating that haploinsufficiency is not causative for UPR stress and therefore not causative for the disease phenotype. In addition, the dispensability of COL X in hypertrophic differentiation and endochondral ossification suggests that silencing the transcription of *COL10A1* gene, including that in the wild‐type locus, can be a therapeutic approach for SMCD.

A PCA of RNA sequencing data showed clear differences between parental and *COL10A1* ^−/−^ clones, but no particular gene sets saw their expression significantly changed (data not shown). However, the expression of specific genes in chondrocytes at different stages in the proliferating zone was lower in *COL10A1* ^*−/−*^ clones. The expression of *COL10A1* is directly and positively regulated by RUNX2^(^ ^39^, ^40^ ^)^ and directly and negatively regulated by SOX9.^(^ ^41^ ^)^ MATN3 indirectly represses the expression of *COL10A1* gene by binding to BMP2, thereby inhibiting BMP downstream signaling.^(^ ^42^ ^)^ There are, however, no previous reports demonstrating the regulation of proliferating chondrocyte‐related genes by COL X. Histological findings of chondrocytes in pellets at day 42 showed a heterogenous cellular morphology (Fig. 3*B* and Supplemental Fig. S4*B*), indicating the presence of cells at different stages in each pellet. The expression level of proliferating chondrocyte‐related genes decreases during the differentiation process. Therefore, the lower expression of these genes in *COL10A1* ^*−/−*^ pellets may indicate that proliferating chondrocytes in day 42 pellets are fewer in *COL10A1* ^*−/−*^ pellets than in parental pellets. In other words, the differentiation process of *COL10A1* ^*−/−*^ pellets may be accelerated compared with parental pellets. Data from in vivo experiments also suggested this conclusion.

To investigate the effect of COL X deficiency in calcification and ossification, we performed two transplantation experiments using 3D chondrocyte pellets, of which one showed the formation of directional growth plate‐like structures and the other showed random trabecular bone formation. The difference might be due to the different in vivo periods (98 days versus 56 days), but it is more likely due to the difference in the in vitro culture period (day 14 versus day 28). Based on the expression of growth plate–related genes (Fig. 2*C* and Supplemental Fig. S3*C*), cells at day 14 are still in the proliferating stage, and histological data in vivo showed that some cells were still in this stage even 98 days after the transplantation and contribute to the formation of growth plate structures. These observations might support previous reports describing stem cell populations in proliferating chondrocytes.^(^ ^43^, ^44^ ^)^ Although the difference was statistically significant only in the case of 414C2, the proportion of the bone area in each tissue was larger in COL X‐deficient tissues than in parental tissues, suggesting the acceleration of differentiation. The ectopic expression of SOX9 in hypertrophic chondrocytes downregulates the expression of *COL10A1*, resulting in the elongation of the hypertrophic zone and reduction of the proliferating zone,^(^ ^45^ ^)^ which may be caused by the accelerated differentiation. One of the proposed functions of COL X is the compartmentalization of matrix components to regulate proper spatiotemporal differentiation,^(^ ^11^ ^)^ and the loss of COL X may induce an improper distribution of matrix molecules to alter the differentiation process.

Cells at day 28 are already positive for *IHH*, a marker for prehypertrophic chondrocytes and also for *RUNX2*. No proliferating chondrocytes were observed in collected pellets, suggesting that most cells at day 28 in vitro may lose the capacity to proliferate and only the differentiation process continued in vivo. The presence of HNA‐positive osteoblasts and osteocytes in these samples, however, suggested that undifferentiated cells with osteogenic potentials existed in day 28 pellets or that hypertrophic chondrocytes transdifferentiated into osteogenic cells. No clear difference was observed between wild‐type‐ and mutant‐derived pellets in these experiments, suggesting that the effect of COL X loss happens only at early stages.

There are several limitations in our hiPSC differentiation system. Most importantly, it lacks the well‐organized regulatory network in growth plates such as the IHH‐PTHrP pathway,^(^ ^1^ ^)^ which may result in a nonphysiological differentiation process. Mechanical factors, which are another important factor for the metabolic regulation of the growth plate,^(^ ^2^ ^)^ are also missing. Further modifications of our system are required to faithfully recapitulate the physiological process of chondrocyte differentiation.

In conclusion, COL X deficiency is dispensable for columnar differentiation processes including hypertrophic differentiation and the endochondral ossification of chondrocytes induced from hiPSCs. COL X deficiency may have, however, an effect on facilitating the differentiation process through an unknown mechanism. The combination of gene‐edited human iPSCs and in vitro and in vivo assay systems for hypertrophic differentiation and growth plate‐like structures is useful for investigating the role of molecules involved in the differentiation and also the molecular mechanism of hypertrophy.

## Author Contributions

Takeshi Kamakura: Data curation; formal analysis; investigation; methodology; validation; writing‐review and editing. Yonghui Jin: Supervision; validation; writing‐review and editing. Megumi Nishio: Data collection. Sanae Nagata: Data collection. Masayuki Fukuda: Data collection. Liping Sun: Data collection. Shunsuke Kawai: Supervision; writing‐review and editing. Junya Toguchida: Conceptualization; funding acquisition; project administration; resources; supervision; visualization; writing original draft, review and editing.

## Disclosures

All authors declare that they have no conflicts of interest and nothing to disclose.

### Peer Review

The peer review history for this article is available at https://publons.com/publon/10.1002/jbm4.10737.

## Supporting information


**Data S1.** Supporting Information.

## Data Availability

Correspondence and request for materials should be addressed to J.T. RNA sequencing data were deposited in the NCBI's Gene Expression Omnibus (GEO) database (GSE218830).
