# Supplementary material for: Collagen X Is Dispensable for Hypertrophic Differentiation and Endochondral Ossification of Human iPSC‐Derived Chondrocytes
Source: JBMR Plus. 2023 Mar 29;7(5):e10737. doi: 10.1002/jbm4.10737 (PMC10184020; doi:10.1002/jbm4.10737)
Supplement: Supplementary file 1 — Data S1. Supporting Information. [file JBM4-7-e10737-s001.pdf]

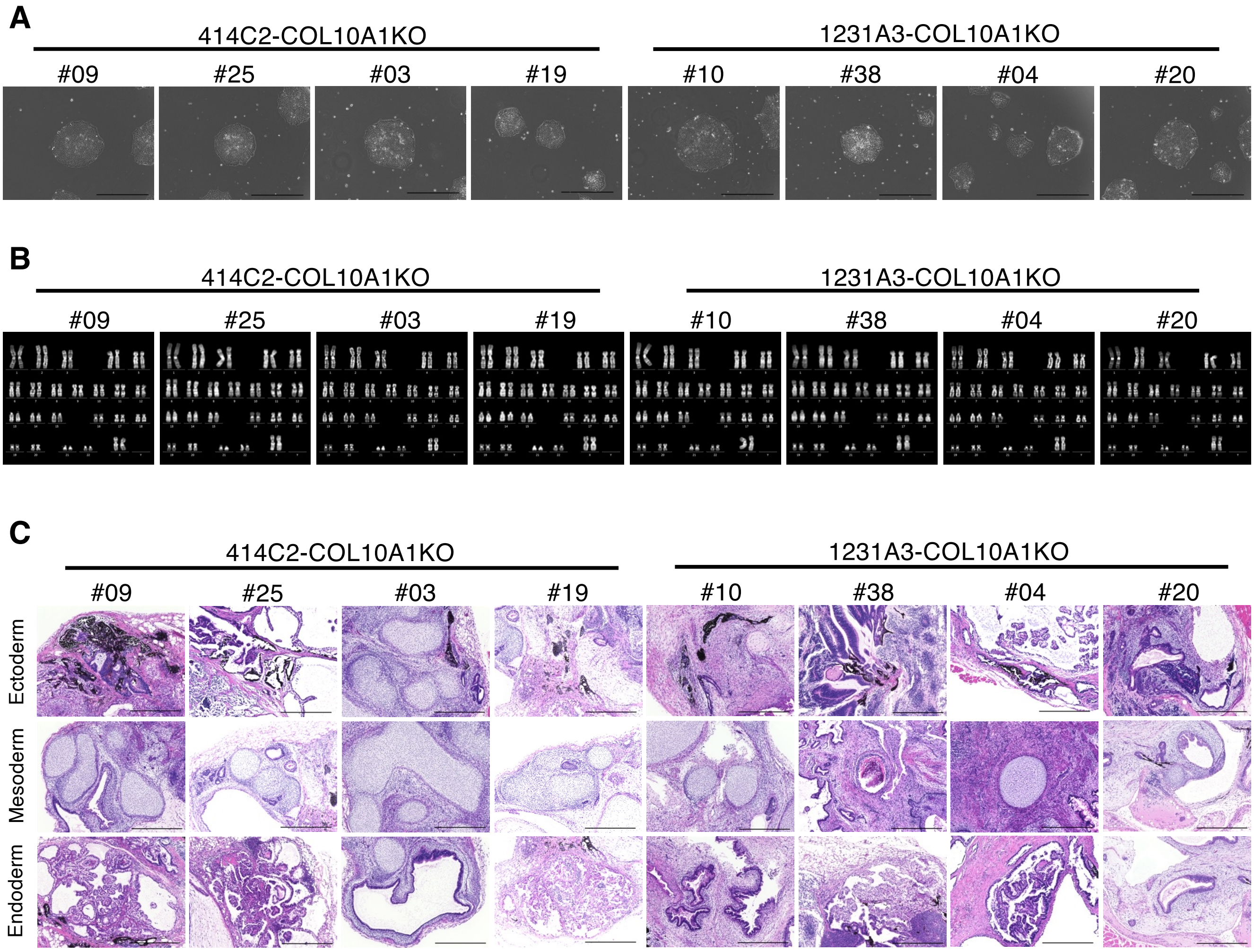

Figure S1. Validation of stem cell features of *COL10A1*$^{+/-}$ and *COL10A1*$^{-/-}$ iPSCs. (*A*) Morphology of colonies by phase contrast images. (*B*) Karyotype by Q-banding. (*C*) Histological findings of three germ layers in teratomas.

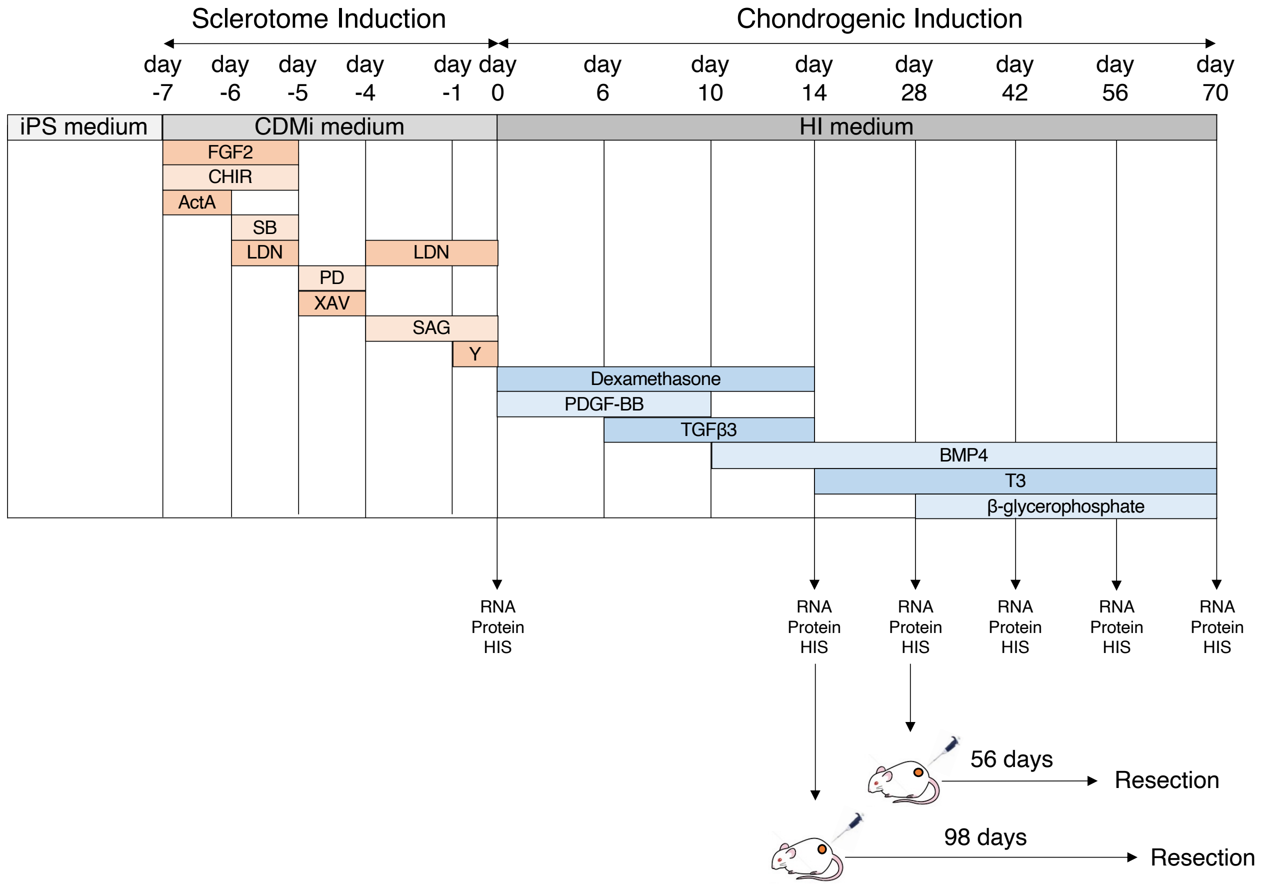

Figure S2. Experimental scheme of the in vitro and in vivo analyses. Chondroprogenitor cells are induced from iPSCs via sclerotome induction and differentiated into proliferating and hypertrophic chondrocytes. Samples are collected at each time point and analyzed. For the in vivo analyses, cellular pellets at day 14 and day 28 are transplanted into immunodeficient mice and collected 98 days and 56 days later, respectively.

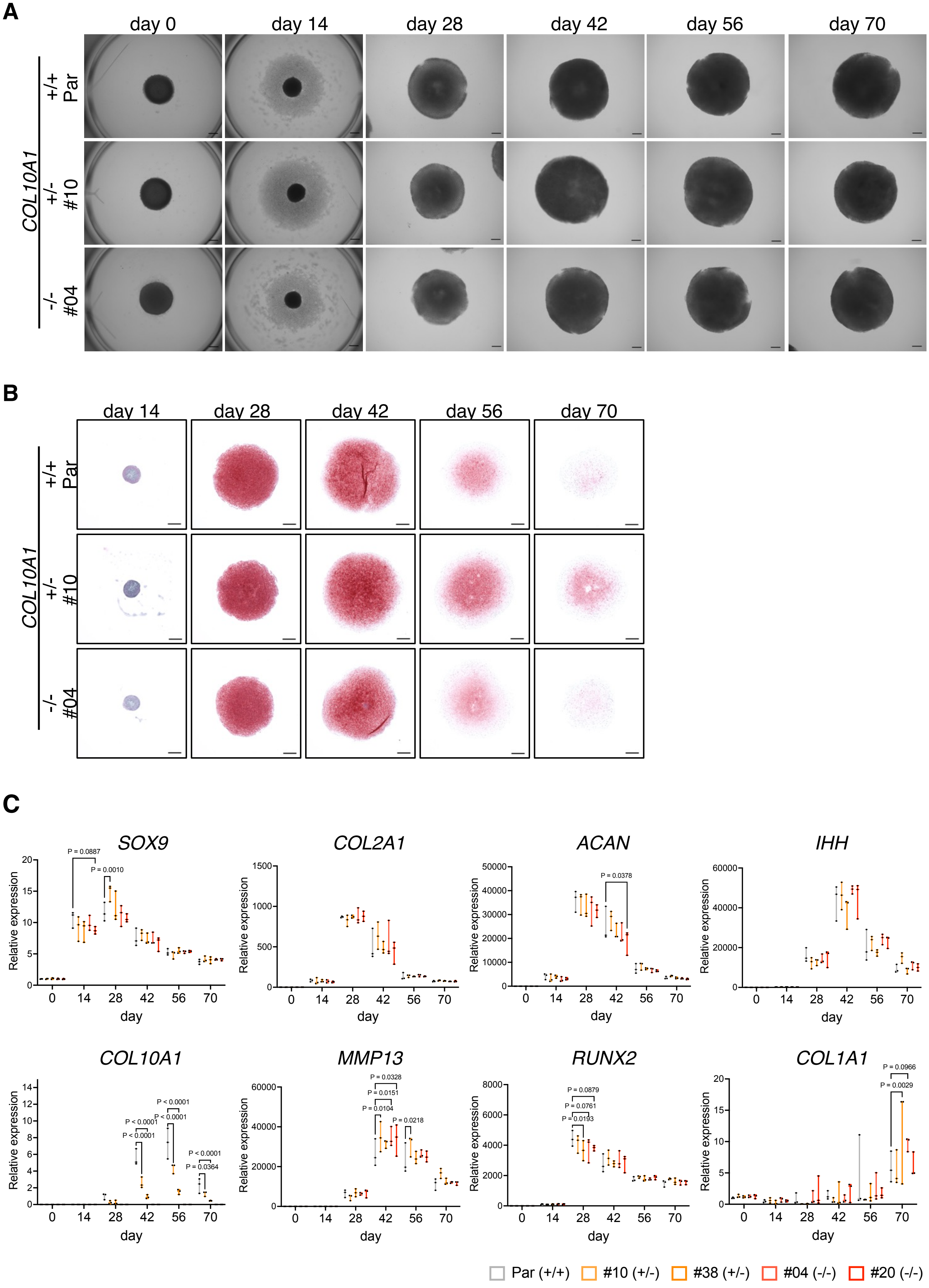
A
B
C
day 0
day 14
day 28
day 42
day 56
day 70
COL10A1
+/+ Par
+/- #10
-/- #04
SOX9
COL2A1
ACAN
IHH
COL10A1
MMP13
RUNX2
COL1A1
Relative expression
day
P = 0.0887
P = 0.0010
P = 0.0378
P < 0.0001
P < 0.0001
P < 0.0001
P < 0.0001
P < 0.0001
P = 0.0364
P = 0.0328
P = 0.0151
P = 0.0104
P = 0.0218
P = 0.0879
P = 0.0761
P = 0.0193
P = 0.0966
P = 0.0029
Par (+/+)
#10 (+/-)
#38 (+/-)
#04 (-/-)
#20 (-/-)

Figure S3. Induction of hypertrophic chondrocytes from parental (1231A3), *COL10A1*$^{+/-}$, and *COL10A1*$^{-/-}$ iPSC lines. (*A*) Phase contrast images and (*B*) Safranin-O and Fast-Green staining of cell pellets at each time point during the induction. Scale bar = 500 μm. Similar results were obtained in three independent experiments. (*C*) mRNA expression of growth plate-related genes during the induction. RNAs were extracted from 3D chondrocyte pellets at each time point and assessed for the expression of each gene by qRT-PCR. The expression level were normalized to those of parental iPSC-derived pellets at day 0, except *COL10A1*, for which the level at day 28 was used for the normalization. Statistical analysis was performed by Two-way ANOVA. Data are presented as boxplots presenting all points from the minimum to maximum values (n = 3, independent experiments).

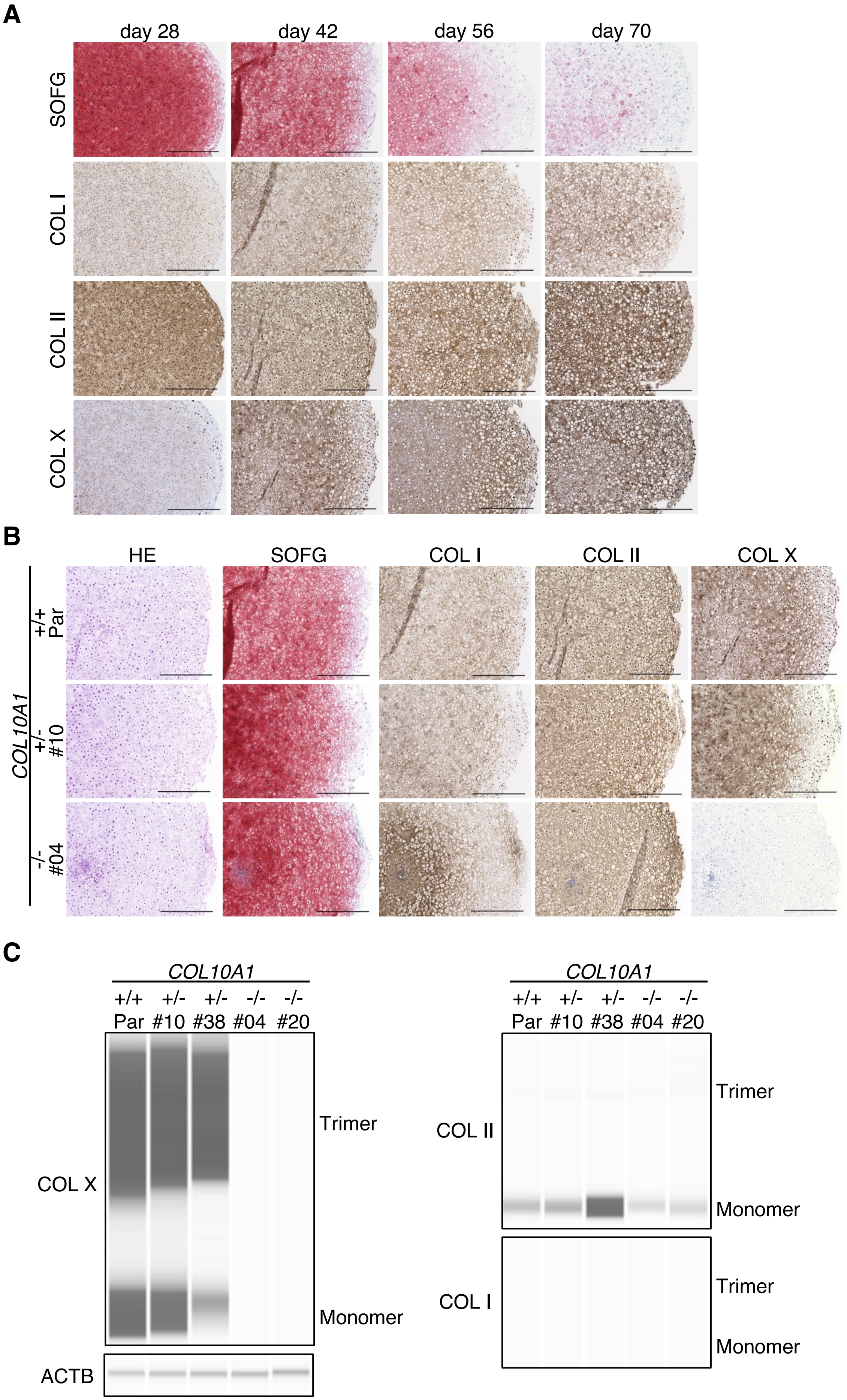
A
day 28
day 42
day 56
day 70
SOFG
COL I
COL II
COL X
B
HE
SOFG
COL I
COL II
COL X
COL10A1
+/+ Par
+/- #10
-/- #04
C
COL10A1
+/+ Par
+/- #10
+/- #38
-/- #04
-/- #20
COL X
Trimer
Monomer
ACTB
COL II
COL I
Trimer
Monomer

Figure S4. Expression of cartilage matrix at day 42 in parental (1231A3), *COL10A1*$^{+/-}$, and *COL10A1*$^{-/-}$ iPSC-derived 3D chondrocytes pellets. (A) Histological evaluation of parental iPSC-derived pellets. Cell pellets at each point were stained with Safranin-O and Fast-Green (SFOG), antibodies against COL I, COL II, and COL X. Scale bar = 500 µm. (B) Histological evaluation of parental, *COL10A1*$^{+/-}$, and *COL10A1*$^{-/-}$ iPSC derived pellets at day 42. Cell pellets were stained with Hematoxylin-Eosin (HE), SOFG, and antibodies against COL I, COL II, and COL X. Scale bar = 500 µm. (C) Capillary-based immunoassay of protein extracted from cell pellets. The proteins were extracted from the pellets at day 42 and analyzed by Wes using antibodies against COL I, COL II, and COL X. The experiments were performed three times with similar results.

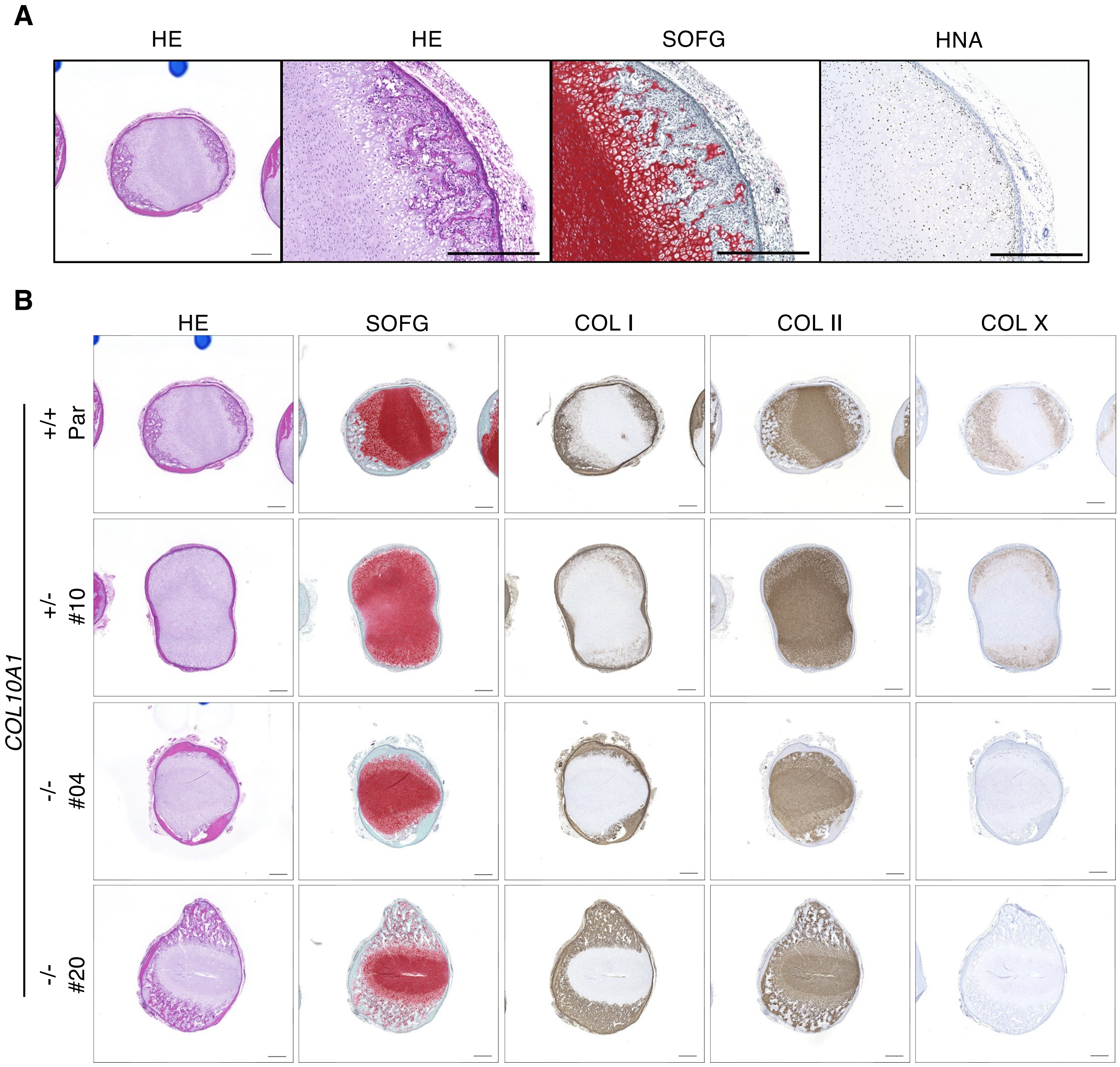

Figure S5. Evaluation of chondrocyte pellets at the proliferating stage in vivo. Cell pellets derived from parental (1231A3), *COL10A1*$^{+/-}$, and *COL10A1*$^{-/-}$ iPSC lines at day 14 were transplanted into immunodeficient mice and collected 98 days later. (*A*) Histology of parental iPSC-derived transplants. The collected samples were stained with hematoxylin-eosin (HE), Safranin-O and Fast-Green (SOFG), or an antibody against human nuclear antigen (HNA). Scale bar = 500 μm. (*B*) histology of parental, *COL10A1*$^{+/-}$, and *COL10A1*$^{-/-}$ iPSC-derived transplants. The collected samples were stained with HE, SOFG, and antibodies against COL I, COl II, or COL X. Scale bar = 500 μm. Similar results were obtained in three independent experiments.

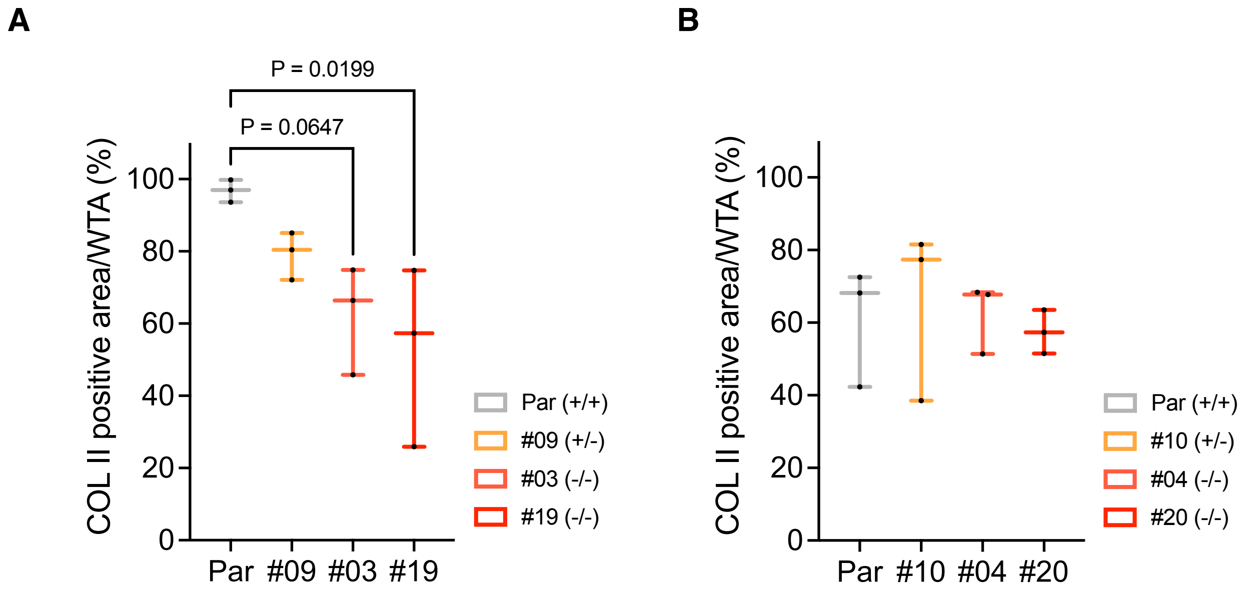

Figure S6. Comparison of COL II positive area between parental (*A*, 414C2; *B*, 1231A3) and mutant iPSC-derived transplants. The COL II positive area in each samples shown in Figure 4*D* (*A*) and S5*B* (*B*) were quantified by the Hybrid Cell Count Module as described in the Methods. Areas relative to the whole tissue area were shown. Data are presented as boxplots presenting all points from minimum to maximum values (n = 3, independent experiments).

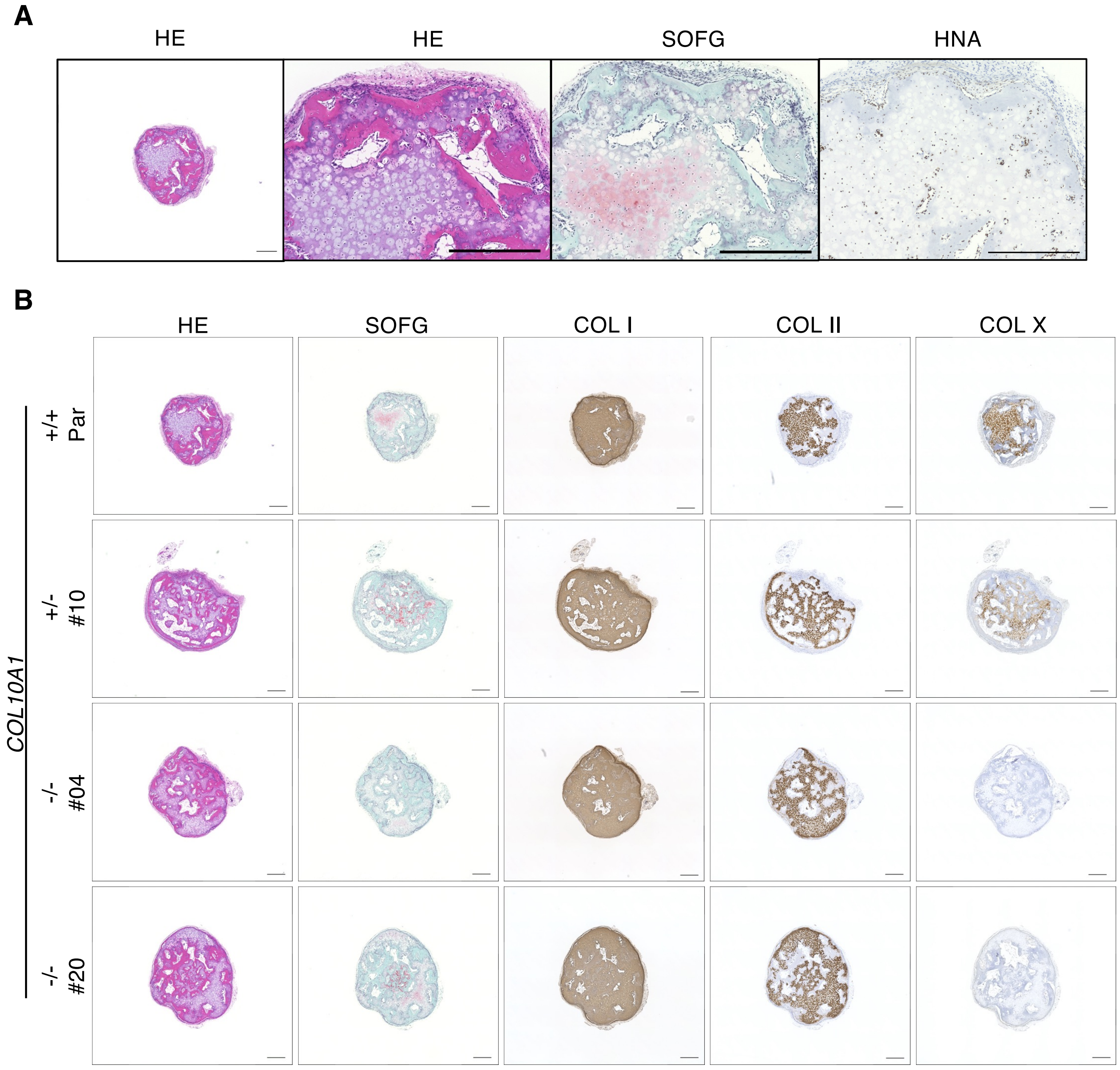

Figure S7. Evaluation of chondrocyte pellets at the prehypertrophic stage in vivo. Cell pellets derived from parental (1231A3), *COL10A1*$^{+/-}$, and *COL10A1*$^{-/-}$ iPSC lines at day 28 were transplanted into immunodeficient mice and collected 56 days later. (*A*) Histology of parental iPSC-derived transplants. The collected samples were stained with hematoxylin-eosin (HE), Safranin-O and Fast-Green (SOFG), or an antibody against human nuclear antigen (HNA). Scale bar = 500 µm. (*B*) Histology of parental, *COL10A1*$^{+/-}$, and *COL10A1*$^{-/-}$ iPSC-derived transplants. The collected samples were stained with HE, SOFG, and antibodies against COL I, COL II, or COL X. Scale bar = 500 µm. Similar results were obtained in three independent experiments.

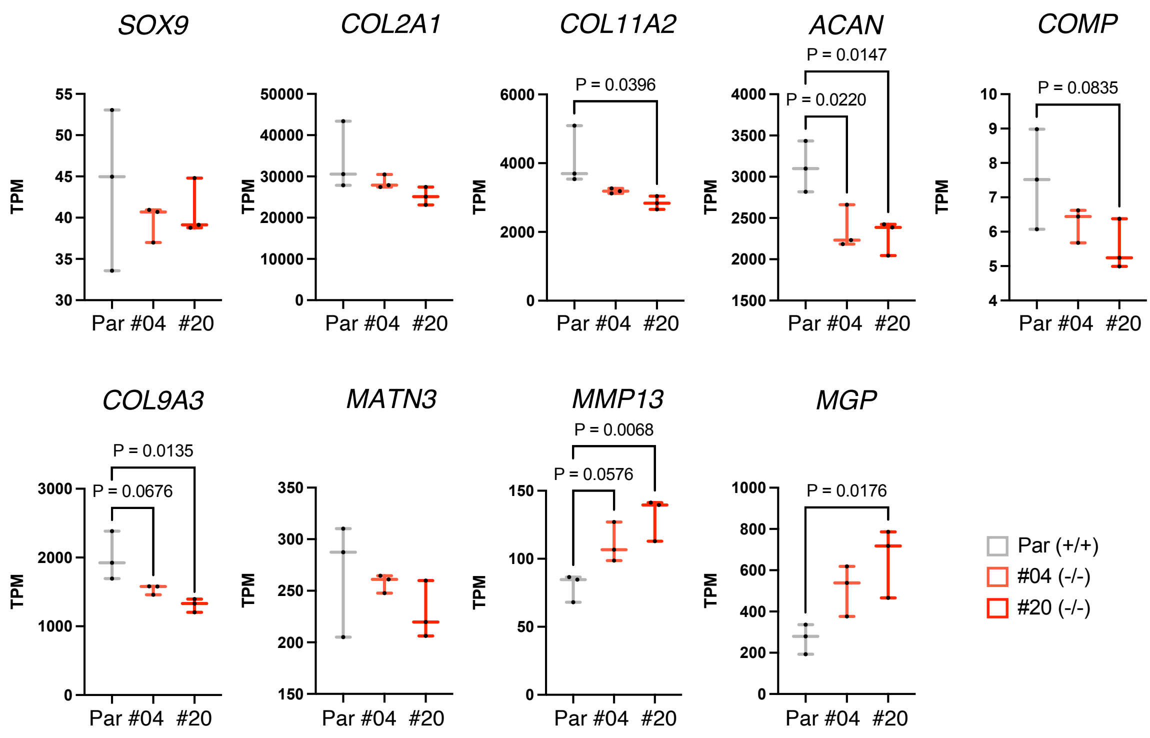

Figure S8. mRNA expression levels of growth plate-related genes in parental (1231A3) and *COL10A1*$^{-/-}$-derived pellets. RNAs were extracted from parental and *COL10A1*$^{-/-}$ iPSCs-derived pellets at day 42 and processed for RNA sequencing analyses. The expression level of each gene is shown as TPM. Data are presented as boxplots presenting all points from minimum to maximum values (n = 3, independent experiments).

Supplementary Table S1. Primers for establishment of hiPSCs lacking *COL10A1* gene

Application	Target region	Direction	Sequence
sgRNA	*COL10A1* N-terminus	Sense	caccTAGGCACACAAGATCGGGCT
		Antisense	aaacAGCCCGATCTTGTGTGCCTA
	COL10A1 C-terminus	Sense	caccAATGACCCTCGTGGCAGGCG
		Antisense	aaacCGCCTGCCACGAGGGTCATT
PCR	*COL10A1* N-terminus	Forward	ATCATTCCACCGTGAACCAG
		Reverse	GGCATTTGGTATCGTTCAGC
	COL10A1 C-terminus	Forward	TCAGGACAATGTGGCTCAAG
		Reverse	CAGTCTTCTTAGTCCCATTGATGC
Seq	*COL10A1* N-terminus	Forward	ATCATTCCACCGTGAACCAG
		Reverse	GCAACAGCATTATGACCCAAGG
	COL10A1 C-terminus	Forward	CCCAGCCGCATTTTTCACATC
		Reverse	CCTACTGTTGGGCAAAGTCATC

Supplementary Table S2. Primers for qRT-PCR

Target gene	Direction	Sequence
ACTB	Forward	CACCATTGGCAATGAGCGGTTC
	Reverse	AGGTCTTTGCGGATGTCCACGT
SOX9	Forward	GACTTCCGCGACGTGGAC
	Reverse	GTTGGGCGGCAGGTACTG
COL2A1	Forward	CGAGGCAACGATGGTCAGCC
	Reverse	TGGGGCCTTGTTCACCTTTGA
ACAN	Forward	TCGAGGACAGCGAGGCC
	Reverse	TCGAGGGTGTAGCGTGTAGAGA
IHH	Forward	CGGTGGACATCACCACATCA
	Reverse	CGTGGGCCTTTGACTCGTAA
COL10A1	Forward	CCCAGCACGCAGAATCCATC
	Reverse	AGTGGGCCTTTTATGCCTGT
MMP13	Forward	CATGAGTTCGGCCACTCCTT
	Reverse	CCTGGACCATAGAGAGACTGGA
RUNX2	Forward	TTACTTACACCCCGCCAGTC
	Reverse	TATGGAGTGCTGCTGGTCTG
COL1A1	Forward	GGACACAGAGGTTTCAGTGGT
	Reverse	GCACCATCATTTCCACGAGC

Supplementary Table S3. Antibodies for Flow cytometry, Immunohistochemistry and Wes

Epitope/Antigen or Product name	Source and Catalog # or RRID	Host species	Application	Dilution	Application specific details
APC-conjugated DLL1 antibody	R&D, FAB1818A	Mouse	FACS	1:200	0.1% BSA in PBS, 30mins at 4°C
APC-conjugated mouse IgG2B	R&D, IC0041A	Mouse	FACS	1:200	0.1% BSA in PBS, 30mins at 4°C
Collagen X Monoclonal Antibody (X53),	Invitrogen,14-9771-82	Mouse	IHC	1:400	1% BSA in PBS, overnight at 4°C
Anti-Nuclei Antibody, clone 3E1.3	Sigma-Aldrich, MAB4383	Mouse	IHC	1:250	1% BSA in PBS, overnight at 4°C
Goat Anti-Type I Collagen-UNLB	SouthernBiotech, 1310-01	Goat	IHC	1:500	1% BSA in PBS, overnight at 4°C
Goat Anti-Type II Collagen-UNLB	SouthernBiotech, 1320-01	Goat	IHC	1:600	1% BSA in PBS, overnight at 4°C
b-Actin (13E5) Rabbit mAb	CST, #4970	Rabbit	Wes	1:200	Antibody Diluent II, 1hrs RT
Collagen I alpha 1 Antibody	Novus, NBP1-30054	Rabbit	Wes	1:50	Antibody Diluent II, 1hrs RT
Collagen II Antibody (5B2.5)	Novus, NB600-844	Mouse	Wes	1:50	Antibody Diluent II, 1hrs RT
Anti-Collagen X antibody [EPR13044]	abcam, ab182563	Rabbit	Wes	1:100	Antibody Diluent II, 1hrs RT

Supplementary Table S4. Biological Modulators for induction of hypertrophic chondrocytes

Modulator	Source, Catalog # or RRID	Solvent/Vehicle	Concentration
BSA	SIGMA, A8806	Media	5 mg/mL
CD lipid concentrate	gibco, 11905-031	Media	1% (v/v)
Apo transferrin	SIGMA, T1147	Media	15 µg/mL
1-Thioglycerol	SIGMA, M6145	Media	450 µM
Insulin	Wako, 097-06474	4 mM HCl	7 µg/mL
rhFGF2	Wako, 068-04544	0.1%BSA	20 ng/mL
CHIR99021	AXON, Axon1386	DMSO	3 µM / 10 µM
Activin A	R&D, 338-AC	4 mM HCl with 0.1%BSA	50 ng/mL
SB431542	Selleck Chem, S1067	DMSO	10 µM
LDN193189	Stemgent, 04-0074	DMSO	250 nM / 600 nM
PD173074	Tocris, 3044	DMSO	100 nM
XAV939	Tocris, 3748	DMSO	1 µM
SAG	Calbio, 566661	DW	100 nM
Y-27632	Wako, 034-24024	DW	10 µM
ITS premix	Corning, 354352	Media	1% (v/v)
45% Glucose	SIGMA, G8769	Media	0.15% (w/v)

Supplementary Table S4. Biological Modulators for induction of hypertrophic chondrocytes (Continued)

Modulator	Source, Catalog # or RRID	Solvent/Vehicle	Concentration
Sodium pyruvate	SIGMA, S8636	Media	1 mM
GlutaMAX-I	gibco, 35050	Media	2 mM
L-ascorbic acid 2-phosphate	SIGMA, A8960	DW	170 µM
Proline	SIGMA, P-5607	DW	350 µM
Dexamethasone	Wako, 047-18863	DW	100 nM
PDGF-BB	R&D, 520-BB	4 mM HCl with 0.1%BSA	40 ng/mL
TGFβ3	R&D, 243-B3	4 mM HCl with 0.1%BSA	10 ng/mL
BMP4	R&D, 314-BP	4 mM HCl with 0.1%BSA	50 ng/mL
triiodothyronine (T3)	SIGMA, T-074	Methanol	10 nM
β-glycerophosphate	SIGMA, G6501	DW	10 mM

Supplementary Table S5. Human iPSC lines

Cell line	Source and Catalog# or RRID	Providing Laboratory	Species, cell type
414C2	CiRA, CVCL_DP60	NA	Human, feeder free iPS cells
1231A3	CiRA, CVCL_LJ39	NA	Human, feeder free iPS cells

Supplementary Table S6. Animals for transplantation of 3D chondrocyte pellets

Mouse model	Source and Catalog#	Strain	Providing Laboratory
NOD/ShiJic-scidJcl	CLEA Japan, NOD/ShiJic-scidJcl	NOD.CB17-Prkdcscid/Jcl	NA
